# Ultrasonically-Assisted and Conventional Extraction from *Erodium Glaucophyllum* Roots Using Ethanol:Water Mixtures: Phenolic Characterization, Antioxidant, and Anti-Inflammatory Activities

**DOI:** 10.3390/molecules25071759

**Published:** 2020-04-10

**Authors:** Francisco J. Barba, Cristina Alcántara, Radhia Abdelkebir, Christine Bäuerl, Gaspar Pérez-Martínez, Jose M. Lorenzo, María Carmen Collado, Jose V. García-Pérez

**Affiliations:** 1 Nutrition and Food Science Area, Preventive Medicine and Public Health, Food Science, Toxicology and Forensic Medicine Department, Faculty of Pharmacy, Universitat de València, Avda, Vicent Andrés Estellés, s/n, Burjassot, 46100 València, Spain; abdelkebirradhia@yahoo.fr; 2 Department of Biotechnology, Institute of Agrochemistry and Food Technology, Spanish National Research Council (IATA-CSIC), Av. Agustin Escardino 7, 46980 València, Spain; crisalba@iata.csic.es (C.A.); cbauerl@iata.csic.es (C.B.);; 3 Range Ecology Laboratory, Institute of Arid Regions (IRA), University of Gabès, Medenine 4100, Tunisia; 4 Centro Tecnológico de la Carne de Galicia, Adva. Galicia n°4, Parque Tecnológico de Galicia, San Cibrao das Viñas, 32900 Ourense, Spain; jmlorenzo@ceteca.net; 5 Grupo de Análisis y Simulación de Procesos Agroalimentarios (ASPA), Departamento de Tecnología de Alimentos, Universitat Politècnica de València, Cami de Vera s/n, 46022 Valencia, Spain; jogarpe4@tal.upv.es

**Keywords:** ultrasound, solid-liquid extraction, polyphenol, antioxidant, anti-inflammatory

## Abstract

The paper presents experimental results concerning the ultrasonically-assisted extraction of bioactive compounds from *Erodium glaucophyllum* roots. A comparison with conventional methodology is presented, and thereby the phytochemical composition and the antioxidant and anti-inflammatory activities of extracts are evaluated. The phenolic profile of *Erodium* extracts was analyzed by TOF–LC–MS–MS. The identification of phenolic compounds revealed that the major component was (+)-gallocatechin in the aqueous extracts obtained for the different extraction methodologies. The highest quantity of phenolic compounds and antioxidant capacity was found in the hydroethanolic extract obtained by conventional extraction (29.22–25.50 mg GAE/g DM; 21.174 mM Trolox equivalent). The highest content of carotenoids, varying from 0.035 to 0.114 mg/g dry matter, was reached by ultrasonic-assisted extraction. Furthermore, *Erodium* extracts showed a potent inhibition of the inflammatory reaction by means of the inhibition of tumor necrosis factor-alpha (TNF-α). The extracts obtained when ultrasound extraction was combined with ethanol:water (50:50, v/v) presented the greatest inhibition (92%).

## 1. Introduction

*Erodium glaucophyllum* belongs to a genus of flowering plants which have been used from ancient times in traditional medicine due to their health attributes. Some of these beneficial properties have been linked to their high content of antioxidant bioactive components, such as vitamin C, polyphenols and carotenoids, among others. For instance, due to the interesting phytochemical profile of *E. glaucophyllum*, several attempts have been made, especially in North Africa, to evaluate its potential use in the areas of agri-food, pharmaceutics, and cosmetics [1,2].

In this vein, Munekata et al. [1] and Al-snafi [3] reviewed the phytochemical profile of *Erodium sp.* showing the potential of *Erodium cicutarium* as a source of vitamin C and phenolic compounds (eg., tannins, catechins, gallic and ellagic acids), with antibacterial and antiviral, antioxidant, and anti-inflammatory properties. Moreover, they found a significant quantity of sugars, amino acids, and vitamin K, among others. In addition, the biochemical composition and the biological activities of *Erodium glaucophyllum* have also been evaluated. Indeed, Bouaziz et al. [4] observed that the aerial part of *Erodium glaucophyllum* presented the highest levels of total phenols and flavonoids after evaluating the phytochemical composition of 25 plants from southern Tunisia, as well as powerful antioxidant activity.

Therefore, there is a need to develop fast, reliable, and efficient methods with which to obtain the extracts. Conventional solid–liquid extraction techniques, involving the use of maceration or Soxhlet, have significant drawbacks, mainly high energy consumption and the use of polluting solvents [5]. Currently, the use of ecological, sustainable extraction methods for natural products is a live issue in the multidisciplinary areas of applied chemistry, biology, and technology. 

Ultrasonic-assisted extraction (UAE) is a promising strategy for the purposes of recovering bioactive molecules [6,7,8]. Literature reports that efficient UAE mainly contributes to the shortening of the extraction times, the use of milder temperatures, and the reduction of solvents; all these aspects not only positively impact on the industrial productivity but also have environmental benefits linked to energy and solvent reduction. In addition, UAE could modify the profile of the extracted compounds compared to conventional extraction since it may facilitate the release of molecules strongly attached to the solid matrix. The effects of UAE are mainly attributed to cavitation phenomena, thus promoting stirring, and also to the temperature increase associated with the ultrasound process; this both promotes bioactive diffusion and solubility, and subsequently the rate extraction yield, and shortens the extraction time [9,10,11,12]. However, it should be noted that when extreme temperatures are reached, the degradation of thermolabile compounds could be promoted [13]. The thermal effect linked to UAE in a liquid medium is frequently not considered during experimentation, which leads to a misunderstanding of the phenomena involved due to the fact that most of the observed effects on the kinetics, yields, or extract composition are brought about by the temperature increase in the medium. In addition, the UAE is often compared to static maceration which evidences a marked effect of ultrasound on fluid turbulence, although similar findings could be reached using efficient mechanical agitators. Therefore, in order to assess the feasibility of UAE compared to other efficient approaches, experimental setups should incorporate accurate temperature control systems and fluid stirrers. However, these aspects are sometimes not taken into account in the available literature.

The process of inflammation is explained according to the production of nitric oxide (NO) from L-arginine by the inducible NO synthase (iNOS). In quick reactions with superoxide, NO gives rise to peroxynitrite. Peroxynitrite is a reactive oxidant and toxic substance that causes tissue damage during inflammatory diseases [14]. Since the critical etiology and exacerbating mechanisms are yet to be identified, it is difficult to find a side-effect free “miracle” drug for chronic inflammatory disorders [15]. Therefore, the demand for natural, safe anti-inflammatory agents has increased. Citing the example of currently used steroidal and non-steroidal anti-inflammatory drugs to treat acute inflammatory disorders, these conventional drugs have not been successful in curing chronic inflammatory disorders, such as chronic rheumatoid arthritis and atopic dermatitis [16].

Plants are a potential source of natural bioactive molecules. They are the subject of rigorous scientific studies for their possible use as alternative medicines, especially for the treatment of anti-inflammatory diseases. Among the several groups of natural products present in plants, their anti-inflammatory activity is mainly attributed to the phenolic compounds. Khlifi et al. [17] showed a good correlation between the phenolic content in plant extracts, in particular that of tannins and flavonoids, with the anti-inflammatory activity. Another study confirmed this correlation, indicating that the flavonoids and sterols identified in several plants are responsible for the anti-inflammatory activity [18].

Despite the great importance and significant benefits that North African countries (e.g., Tunisia) could gain from *E. glaucophyllum*, to the best of our knowledge, the literature evaluating the phytochemical profile of *E. glaucophyllum* is scarce. Evaluating the phytochemical profile is a crucial step prior to using a plant. Moreover, there is no information about the best extraction method with which to extract the phytochemical compounds from *E. glaucophyllum* and the potential implications of the anti-inflammatory response. Therefore, this study analyzed the antioxidant profile of *E. glaucophyllum* extracts obtained after conventional or UAE combined with binary mixtures of ethanol and water. Moreover, the anti-inflammatory response of the extracts was evaluated and compared.

## 2. Results and Discussion

### 2.1. Assessment of Total Phenolic Compounds, Total Flavonoids, Phenolic Profile, Carotenoid Content, and Antioxidant Capacity 

**Figure 1:**
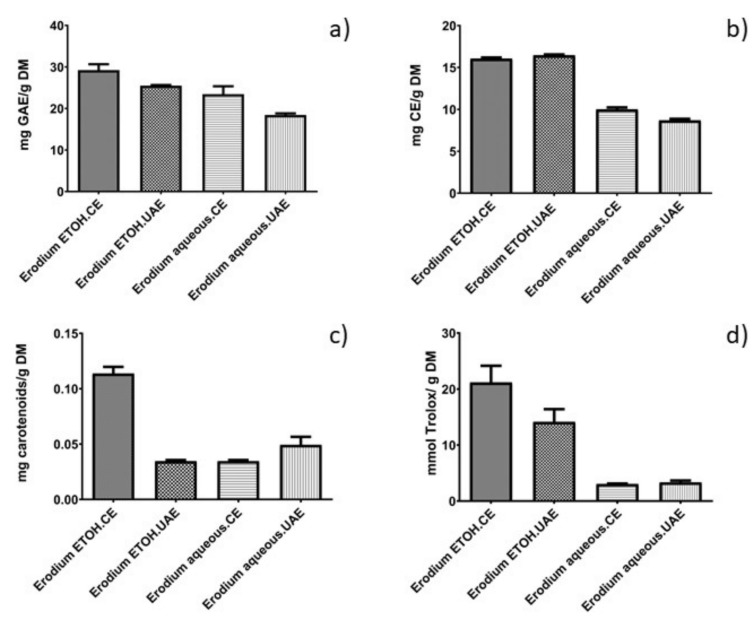
(**a**) The total phenolic compounds expressed as mg gallic acid equivalents (GAE)/g dry matter (DM), (**b**) the flavonoid content expressed as mg catechin equivalents (CE)/g dry matter (DM), (**c**) total carotenoid content expressed as mg carotenoids/g dry matter (DM), and (**d**) antioxidant capacity (TEAC) expressed as mM Trolox equivalents/g dry matter (DM); determined in *Erodium glaucophyllum* in aqueous extract and ethanol (ETOH) extract (50:50, v/v, ethanol:water). UAE: extracts obtained by ultrasound-assisted extraction; CE: extracts obtained by conventional extraction.

Firstly, the contents of the total phenolic compounds (TPC), total flavonoids, carotenoids, and the total antioxidant capacity of the *E. glaucophyllum* hydroethanolic extracts obtained after conventional extraction (CE) or ultrasound-assisted extraction (UAE) were evaluated (Figure 1).

Overall, the total phenolic contents (TPC) were similar to the results previously reported by Bouaziz et al. [4] when they evaluated the TPC composition of aerial parts of *Erodium glaucophyllum* (22.25 mg pyrogallol equivalents/g dry matter (DM)). In this study, the authors compared the levels of the phenolic compounds of 25 plants and found that the extract of the aerial part of *E. glaucophyllum* had the highest content.

Moreover, the ANOVA analysis showed that both treatment and solvent had a significant impact (*p* < 0.05) on TPC *Erodium* extracts. In fact, the extracts obtained after CE exhibited a higher TPC than the UAE treatments (Figure 1). These results are in close agreement with those previously reported by Zhang et al. [19] and Qiao et al. [20] who also observed a significant effect of ultrasound on the degradation of gallic acid and phenolic acids, especially when ultrasound was applied at high energy levels. The authors attributed this phenomenon to the changes in the physicochemical properties of the liquid phase under ultrasonic irradiation, commonly having significant effects on i) the number of the cavities or ii) the pressure/temperature pulse generated, which are attributed to the collapse of the cavity. This fact could be attributed to the instability of these phenolic compounds under UAE conditions (temperature, time, power, solvent). For a detailed description about the UAE mechanism see [21,22,23]. In addition, it could be also considered that under these particular conditions, the optimized CE used was more efficient than UAE to release the phenolic compounds from the solid matrix. 

The combination of ethanol:water (50:50, v/v) permitted TPC values to be higher (29.223–25.504 mg GAE/g dry matter (DM)) than aqueous-assisted extraction (23.454–18.445 mg GAE/g DM). Previous studies have shown that polyphenols are compounds of moderate polarity, which tend to be extracted with solvents with medium polarity; thus, ethanol is a more convenient solvent than water with which to extract TPC. Similar findings were also reported by Wei et al. [24] after using solvents of increasing polarities for the characterization of medicinal plants and Carbonell-Capella et al. [25] when comparing combinations of ethanol:water vs. water to extract TPC from *Stevia rebaudiana* Bertoni leaves. Owczarek et al. [26] showed that a polyphenol-rich extract exhibits an inhibitory effect on the insulin-like growth factors that participate in several cellular processes (proliferation, differentiation, and apoptosis).

Like the results obtained for TPC, the ANOVA analysis showed that the total flavonoid content was markedly superior (*p* < 0.05) in hydroethanolic extracts (≈16 mg catechin equivalents (CE)/g DM) than in aqueous extracts (≈9 mg CE/g DM). These values were significantly higher than those reported by Bouaziz et al. [3] when they evaluated the total flavonoid content of *Erodium glaucophyllum* (2.03 mg rutin equivalents/g DM). However, the negative effects of ultrasound were only shown when water was used as a solvent. On the other hand, Galvan D’Alessandro et al. [27] tested the yield of phenolic compounds in black chokeberry extracts obtained following ultrasound extraction, varying the ethanol:water percentages. The authors noted that the presence of ethanol in the extraction solvent increased the yield of polyphenols, and a 50% extraction of ethanol was 2 times higher than the extraction yield with pure water. Solvents with alcoholic percentages have been commonly used to extract phenolic compounds (anthocyanins, flavonoids, tannins, etc.) from plant matrices [28,29].

In order to evaluate the influence of the treatment on the phenolic profile, a triple TOF–LC–MS–MS analysis was carried out (Table 1). As can be seen in Table 1, the profile of individual phenolic compounds obtained from conventional and ultrasound extracts were different. For instance, the analysis of the aqueous extract of *Erodium* obtained following a CE identified 5 flavonoids, 3 phenolic acids, and 4 polyphenols corresponding to the group of “other polyphenols,” while 10 flavonoids, 5 phenolic acids, 1 phenol, 2 polyphenols corresponding to the group of “other polyphenols,” and 1 stilbenoid were identified after UAE. 

The predominant compound for both extracts was (+)-gallocatechin, and the intensity of the peak was higher in the extracts obtained by CE (Table 1). Moreover, our results coincide with those found by Roseiro et al. [30], showing that the phenolic profile of the carob (*Ceratonia siliqua L.*) extracts obtained using CE presented chromatograms with peaks more intense than those obtained after UAE. 

In another study, the UAE of blackberry juice promoted the degradation of the juice’s color, which was mainly due to the degradation of carotenoid and anthocyanin pigments [31]. According to the authors, the degradation under UAE can be caused by cavitation, which governs various reactions: the modification of diffusion rates, the quickening of chemical reactions, and the degradation of enzymes and microorganisms [32]. Da Porto et al. [33] compared the extraction of polyphenols from grape (*Vitis vinifera L.*) seeds using two different methods: CE and UAE. The authors observed that UAE promoted a decrease in the contents of phenolic compounds, such as cinnamic acids, and the degradation of certain compounds [34]. Zhang et al. [19] also observed the same effect of UAE on the degradation of phenolic compounds. This result agrees with the TPC content (Figure 1) which, overall, showed a smaller quantity of phenolic compounds in UAE extracts. Finally, it can be also hypothesized that in the phenolic extraction of polyphenols the UAE was less efficient than the optimized CE employed in this work using mechanical stirrers.

It is difficult to compare the results obtained in our study for *Erodium* as the literature evaluating its phenolic profile is scarce, and there are no studies available reporting the impact of the extraction conditions used in our study. Zbigniew, Sroka, and Mażol [35] studied the phenolic profile of *Erodium cicutarium* by analysis with GC–MS and their antioxidant effect. The main phenolic compounds identified were tannins, gallic acid, and catechin. The presence of these phenolic compounds in the aqueous *Erodium glaucophyllum* extracts confers antibacterial, antiviral, and antifungal activities [36]. Prakash et al. [37] described the biological activities of catechin-based molecules, such as epicatechins. These metabolites extracted from plants demonstrated various biological functions, such as anti-proliferative, anti-inflammatory, antioxidant, antimicrobial, and cardio-protective activities.

Carotenoid content is of interest due to their potential use not only as natural pigments but also as antioxidants. For instance, the content varied from 0.114 to 0.035 mg/g dry matter, with the highest quantity of carotenoids obtained after UAE, combined with the mixture ethanol:water (50:50, v/v), while the conventional aqueous extraction presented the lowest carotenoid yields. These results can be explained by the fact that cavitation linked to UAE facilitated the penetration of the solvent through the cell membrane into the plant matrix and, as a consequence, more pigments were extracted than during CE [25,38]. In addition, the increase of turbulence caused by UAE may also facilitate the diffusion from the solid surface to the bulk solvent. Carbonell-Capella et al. [25] also observed that the hydroalcoholic extracts increased the extraction of carotenoids compared to the aqueous extracts.

In regards to total antioxidant capacity, some previous studies have suggested that phenolic compounds are mainly responsible for the antioxidant activity of plants [39]. This study revealed that the antioxidant activity expressed as the Trolox equivalent antioxidant capacity (TEAC) of *Erodium* hydroethanolic extracts (21.17 mM Trolox equivalent (TE)) was significantly higher than that of the aqueous extracts (3.04 mM TE). Since it is known that the TEAC value quantifies the antioxidant activity, a higher TEAC value characterizes a greater activity of our extracts.

The experimental results revealed that the plant under study clearly exhibited a higher antioxidant activity than some common vegetables and fruits (nutritional plants). Similarly, Bouaziz et al. [4] confirmed that the antioxidant activity of *Erodium glaucophyllum* was dependent on the solvent used, observing that the methanolic extract of *Erodium glaucophyllum* exhibited the highest free radical scavenging activity (DPPH) antiradical power (IC50 = 0.44 μg/mL) of 100 plant extracts [4]. 

### 2.2. Anti-Inflammatory Analysis

**Figure 2:**
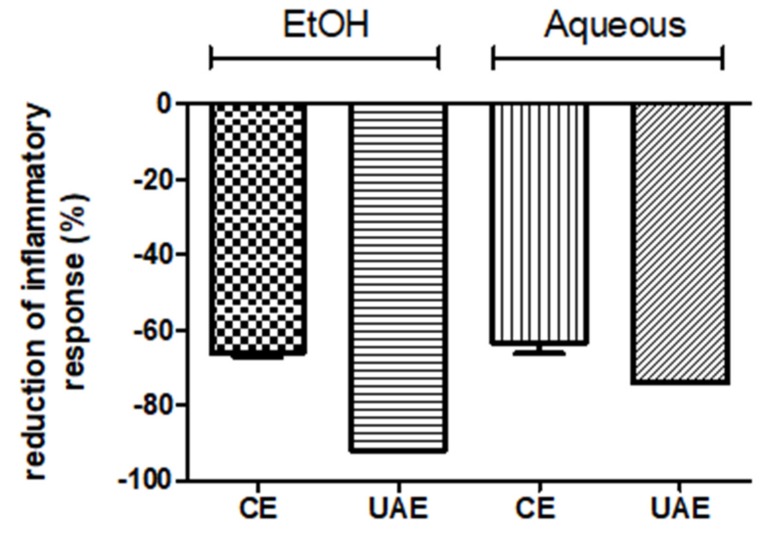
The effect of *Erodium glaucophyllum* extracts on TNF-α-induced pro-inflammatory response. The diagram shows the reduction of secreted alkaline phosphatase activity (SEAP) activity (%) in the cell line reporter (HT-29 clone #16) with respect to TNF-α (0%). EtOH: hydroethanolic extract; aqueous: extracts with water. UAE: extracts obtained by ultrasound-assisted extraction; CE: extracts obtained by conventional extraction.

Finally, the potential of *Erodium* extracts for anti-inflammatory response was determined by measuring the inhibition of tumor necrosis factor-alpha (TNF-α)-induced pro-inflammatory gene expression in HT-29 human colon cells (Figure 2). 

As can be seen in Figure 2, both treatment and solvent had a significant effect on the anti-inflammatory response of the extracts obtained. The ANOVA analysis showed significant differences between the extracts obtained following different extraction methods and by using different extraction solvents. 

As far as UAE is concerned, the extracts obtained when the UAE was combined with ethanol:water (50:50, v/v) presented the greatest inhibition (92%) of the inflammatory response followed by the aqueous extract (72%). Similar behavior was also found in the case of the conventional extraction, obtaining the highest inhibition percentage (65%) after using the hydroethanolic mixture compared to the aqueous extraction (63%).

The significant anti-inflammatory activity of *Erodium glaucophyllum* could be essentially attributed to the presence of phenolic compounds. Along these lines, the antibacterial activity of Geraniaceae genera was evaluated in a previous study, and the authors observed an anti-inflammatory potential of the extracts obtained due to their ability to modulate pro-inflammatory cytokine expression [40]. Moreover, some compounds from *Erodium* have been involved in inhibiting the formation of 5-lipoxygenase, which is associated with the inflammatory response [41]. 

Choi et al. [42] described the potent effect of phenolic compounds and terpenoids as anti-inflammatory agents. For this reason, numerous plants rich in potent antioxidant compounds have been exploited in the treatment of inflammatory diseases. These results agree closely with those previously reported by Asif and Khodadadi [43], who pointed out the important role of flavonoids in anti-inflammatory activity. As was previously indicated, several studies indicated that *Erodium* extracts had important levels of phenolic compounds [4]. As shown in [Table molecules-25-01759-t001], the *Erodium* extracts contain isorhamnetin 3-*O*-glucoside, which is a quercetin derivative. A recent study showed that this compound has a significant inhibitory effect on the synthesis of inflammatory mediators (12-HHT, TXB_2_, and PGE_2_), comparable to aspirin [44].

## 3. Materials and Methods 

### 3.1. Chemicals and Reagents

Aluminum chloride solution, 2,2′-azinobis-(3-ethylbenzothiazoline-6-sulfonic acid) diammonium salt, ABTS radical (≥99% purity), (+)-catechin, Folin–Ciocalteu reagent, 6-hydroxy-2,5,7,8-tetramethylchroman-2-carboxylic acid, gallic acid, HPLC-grade solvents (acetonitrile, methanol, and formic acid), Trolox^®^ (97% purity), resveratrol, and potassium persulfate were purchased from Sigma–Aldrich (St. Louis, MO, USA). Disodium hydrogen phosphate, potassium dihydrogen phosphate, sodium bicarbonate, sodium carbonate, chelex^®^ dipotassium peroxodisulfate, and absolute ethanol were obtained from Merck (Barcelona, Spain), whereas chloride acid (37%) was supplied by Merck and Co., Inc. (Whitehouse Station, NJ, USA). 

### 3.2. Plant Material

*Erodium glaucophyllum L.* was collected from the south east of Tunisia (Boughrara), in the flowering stage (April 2015). After the separation of the root bark, the plant material was washed and air-dried at room temperature (25 °C) to a constant weight. Afterwards, it was ground (Blixer 2, Robot Coupe, France) to a uniform powder and particles of under 0.05 mm were used. Finally, the powder was vacuum packaged and stored at 4 ± 1 °C in the dark until needed. The authenticity of the plant materials was confirmed by the evaluation of the morphological structure of the samples made by experts in the Department of Plant Biology of the University of Valencia (Valencia, Spain).

### 3.3. Extraction Experiments: Ultrasound-Assisted (UAE) and Conventional (CE) Extraction

The experiments were performed according to the set-up previously established by Khemakhem et al. [11]. UAE experiments were conducted using an ultrasonic probe system (UP400S, Dr. Hielscher, Teltow, Germany). The ultrasonic emitter, with a diameter of 2.2 cm, was placed in a jacketed vessel and immersed 1 cm in the solvent. The extraction temperature was controlled by re-circulating an ethylene glycol solution (40%) through a cooling coil made with a copper tube (4 mm diameter), which was immersed in the solvent, and also through the jacket of the vessel. In order to re-circulate the ethylene glycol solution, a peristaltic pump (302 S, Watson–Marlow, Postfach, Germany) was connected to a cooling reservoir (refrigerated Circ, Model 1190S, USA) and to a process controller (E5CK, Omron, Hoofddorp, Netherlands) to perform an ON–OFF type control. The temperature of the solvent was monitored using a Pt100 sensor, which was also wired to the controller.

Extraction experiments were carried out using distilled water (100%, v/v) or a hydroethanolic solution (50%, v/v) as solvents. A ratio of 2% (w/v, weight of dry root/volume of water) and a total volume of 100 mL were used for each experiment. The experiments were performed at a constant temperature of 40 ± 1 °C, supplying 100% of the total power of the system (300 W) in every case. The extraction time was fixed at 10 min according to the optimal conditions previously established by other authors [11]. Once the sample was taken, the same volume of water was introduced to keep the ratio of dry roots/volume of solvent constant. Each extract was filtered (0.45 μm) prior to the analytical determinations and the extraction experiments were performed in triplicate.

The characterization of the ultrasonic field was determined by a calorimetric method, used to estimate the actual ultrasonic power released into the medium. To achieve this purpose, the temperature of the solvent was measured every second for the first 3 min of the ultrasonic application without the sample and without controlling the temperature. Then, the ultrasonic power applied (*P* [W]) was calculated using the rise in temperature, as expressed in Equation (1):

(1) $$P=mC_{p}\frac{dT}{dt},$$

where *m* (kg) is the solvent mass, *C_p_* (J/kg °C) the specific heat of the solvent, and *dT/dt* is the slope of the logged temperature–time curve.

The ultrasonic power was measured, in triplicate at least, using two type-K thermocouples wired to a data logger. In this case, the effective ultrasonic power measured was 32.2 ± 1.5 W, by providing 300 W of electrical energy to the transducer.

CE experiments were carried out in the same experimental conditions, but replacing the ultrasonic probe by a mechanical stirrer (RZR 1, Heidolph, Germany) provided with an axial flow impeller working at 540 rpm. The same control temperature system as in the UAE experiments was used, but with the control loop working in heating mode (set point 40 °C).

### 3.4. Total Polyphenol Content and TOF–LC–MS–MS Analysis

The total amount of polyphenols was measured spectrophotometrically by the Folin–Ciocalteu method, based on a colorimetric oxidation/reduction reaction of phenols [45] with some modifications to prevent the interferences that are common in the traditional Folin-Ciocalteau method. Two hundred microliters of diluted extract and 1 mL of Folin–Ciocalteu reagent (diluted 1:10 with water) were mixed. Then, 0.8 mL of Na_2_CO_3_ (75 g/L) was added. The sample was incubated for 10 min at 50 °C and then cooled to room temperature. For the control sample, 0.2 mL of distilled water was taken. The absorbance was measured at 750 nm by the UV/Vis spectrophotometer (Libra S32, Biochrom, France). Gallic acid was used for the calibration curve. The results were expressed as mg gallic acid equivalents (GAE)/g dry matter (DM). The analyses were performed in triplicate and the average deviation was calculated.

In order to determine total flavonoids, the method of Sakanata et al. [46] was used. Two hundred and fifty microliters of extract (appropriately diluted) were mixed with 1.25 mL of distilled water and 75 μL of a sodium nitrite solution (5%, w/v), and left to rest for 6 min. Then, 10% aluminum chloride solution (150 μL) was added and kept for 5 min under resting conditions. Afterwards, 500 µL of sodium hydroxide (1 M) were added and the mixture was brought to 2.5 mL using distilled water and mixed. The absorbance was measured at 510 nm in a UV/Vis spectrophotometer. (+)-Catechin was used to prepare a calibration curve, and the results were expressed as mg catechin equivalents (CE)/g DM. 

The identification of the major phenolic compounds present in the *Erodium glaucophyllum* extracts was carried out using a TripleTOF™ 5600 (AB SCIEX) LC–MS–MS system equipped with Agilent 1260 Infinity (Agilent, Waldbronn, Germany) according to the method previously published of Abdelkebir et al. [47]. Chromatographic separation was performed using a Waters UPLC C18 column 1.7 µm (2.1 × 50 mm) Acquity UPLC BEH.C18 from Waters (Cerdanyola del Vallès, Spain). The mobile phase was composed of water (0.1% formic acid, A) and methanol (0.1% formic acid, B). The gradient elution of the mobile phase was as follows: from 0 to 13 min, 0%–90% B (v/v); 13–15 min, 100% (v/v) B; 15.1–22 min, 90% (v/v) A. The flow rate used was 0.4 ml/min. The injection volume was 5 μL.

The MS acquisition was performed in negative mode, over a mass range of 80–1200 *m*/*z*. An automated calibration was carried out using an external calibration delivery system (CDS), which infuses a calibration solution prior to sample introduction. The MS was done using an IDA acquisition method with the survey scan type (TOF–MS) and the dependent scan type (product ion) using −50V of collision energy. The MS parameters were: ion spray voltage, −4500 V; declustering potential (DP) 90 V; collision energy (CE) −50 V; temperature 400 °C with curtain gas (CU) 25 psi; ion source gas 1 (GC1) 50 psi, and ion source gas 2 (GS2) 50 psi. IDA MS–MS was performed using the following criteria: ions that exceeded 100 CPS, ion tolerance 50 mDa, collision energy fixed at 25 V, and dynamic background subtract activated. Data acquisition and processing were carried out using the software analyst PeakView1.1, with the application XIC Manager and Formula Finder. 

### 3.5. Total Carotenoids

Total carotenoid extraction was carried out in accordance with Lee and Castle [48]. An aliquot of extract (2 mL) was homogenized with 5 mL of extracting solvent (hexane/acetone/ethanol, 50:25:25, v/v) and centrifuged for 5 min at 4000 rpm at 5 °C. The top layer of hexane containing the color was recovered and transferred to a 25 mL volumetric flask and the volume was then adjusted to 25 mL with hexane. Total carotenoid determination was carried out on an aliquot of the hexane extract by measuring the absorbance at 450 nm. Total carotenoids were calculated according to Ritter and Purcell [49] using an extinction coefficient of β-carotene, E^1%^ = 2505.

### 3.6. Trolox Equivalent Antioxidant Capacity (TEAC)

The antioxidant activity of *Erodium* extracts was determined using the TEAC method [50]. Trolox (5 mM) was prepared in ethanol for use as a stock standard. ABTS radical (ABTS^·+^) was generated by mixing 25 mL ABTS and 440 μL of potassium persulfate (140 mM). Then, the mixture was allowed to stand in the dark at room temperature for 16 h before use. The solution was diluted with ethanol until an absorbance of 0.70 ± 0.02 was reached at 734 nm. Once the radical was formed, 2 mL of ABTS^•+^ were mixed with 100 μL of extract (appropriately diluted) and the absorbance was measured at 734 nm for 30 min in a Perkin Elmer UV/Vis Lambda 2 spectrophotometer (Perkin-Elmer, Oberlingen, Germany) in accordance with Barba et al. [51]. The results, obtained from triplicate analyses, were expressed as mM Trolox equivalents (TE).

### 3.7. Anti-Inflammatory Activity

The human colon tumorigenic cell line HT-29 clone #16 (kindly provided by Prof. G. Perez-Martinez, laboratory Lactic Acid Bacteria and Probiotics, IATA-CSIC, Valencia, Spain) contains a secreted alkaline phosphatase (SEAP) reporter gene under the control of the NF-κB promotor after stable transfection with the plasmid pNiFty2-SEAP (Invivogen), in order to detect pro-inflammatory gene expression induced by the transcription factor NF-κB, a master regulator in inflammatory signaling. The cell line was routinely maintained in Dulbecco’s Modified Eagle Medium (DMEM) high glucose supplemented with 10% fetal bovine serum (FBS), 1 mM sodium pyruvate, antibiotics (100 U/mL penicillin, 100 µg/mL streptomycin), and zeocin (200 µg/mL). Cells were cultured at 37 °C in a humidified atmosphere containing 5% CO_2_. 

For each experiment, the cells were detached using 0.25% Trypsin–EDTA solution (Gibco, Life Technologies) resuspended in complete cell culture medium and seeded at 70,000 cells/well in 96-well plates. The cells were grown 24 hours before the experiment and then stimulated simultaneously with TNF-α at final concentration of 10 ng/mL (Immunotools, Friesoythe, Germany) and 10 µL of each extract in a final volume of 100 µL. After 24 hours of stimulation, SEAP (secreted alkaline phosphatase) activity in the cell culture supernatant was quantified using *p*-nitrophenyl phosphate as phosphatase substrate, according to the manufacturer’s instructions (Thermo Scientific, Ref.: 37620). The yellow-colored reaction products were detected using a microplate reader (Multiskan Ascent) at 414 nm.

### 3.8. Statistical Analyses

An analysis of variance (ANOVA) was used in order to analyze the influence of the extraction solvent and mode (UAE and CE methods) on the bioactive compound content, antioxidant capacity and anti-inflammatory parameters. A Tukey’s Multiple Comparison Test was applied to indicate the samples between which differences existed. A multiple regression analysis was performed to study the influence of different factors on a given parameter. [52]. All statistical analyses were performed using the Statgraphics^®^ Centurion XV (Statpoint Technologies, Inc., USA) software.

## 4. Conclusions

From the results obtained in this study, it can be concluded that the solvent (ethanol:water) and extraction methodologies (conventional and ultrasonically-assisted) led to relevant differences in the quantity of bioactive compounds extracted. This study confirmed that the phenolic compounds of *Erodium glaucophyllum* had a higher affinity for the hydroethanolic mixture (50:50, v/v, ethanol:water) than aqueous media. However, significant differences were also observed between conventional and ultrasonically-assisted extracts in terms of total phenolics, flavonoids, and carotenoids. We have demonstrated that the different *Erodium glaucophyllum* extracts are endowed with a potent anti-inflammatory activity and that the hydroethanolic extract obtained by ultrasound extraction has the highest percentage of TNF-α inhibition. The potential of *Erodium* extracts as anti-inflammatory compounds has been shown, although further studies should be conducted to elucidate which specific compounds or group of compounds are mainly responsible for the anti-inflammatory activities. For this purpose, it would be necessary to isolate and purify each specific compound from *Erodium* and evaluate its activity. Finally, further research is necessary in order to bring the extraction of *Erodium glaucophyllum* to an industrial level. Thereby, the performance of conventional and ultrasonic-assisted extraction has to be compared at continuous reactors, instead of the batch-type used in this work, and testing of higher ratios of dried material/solvent, as well as the energy and economic analysis of both strategies, has to be conducted.

## Figures and Tables

**Table 1 molecules-25-01759-t001:** Specific polyphenols (peak intensity) identified in *Erodium glaucophyllum* aqueous extracts confirmed by TOF–LC–MS–MS obtained by conventional extraction (CE) and ultrasound-assisted extraction (UAE).

Group	Compound Name	Score	Formula	Threshold	Expected *m*/*z*	CE (Intensity Average)	UAE (Intensity Average)
**Flavonoids**	(+)-Catechin	91%	C_15_H_14_O_6_	50	289.0718	141,164 ± 29,059	75,051 ± 4815
	(+)-Catechin 3-*O*-glucose	92%	C_21_H_24_O_11_	50	451.1246	-	50,611 ± 5769
	Dihydroquercetin	86%	C_15_H_12_O_7_	50	303.051	-	33,681 ± 2987
	(+)-Gallocatechin	93%	C_15_H_14_O_7_	50	305.0667	382,755 ± 33,378	323,730.5 ± 28,429
	Hydroxytyrosol 1-*O*-glucoside	90%	C_14_H_20_O_9_	50	331.1035	-	28,168 ± 72
	Isorhamnetin 3-*O*-glucoside	89%	C_22_H_22_O_12_	50	477.1039	-	8504 ± 0
	Myricetin 3-*O*-galactoside	90%	C_21_H_20_O_13_	50	479.0831	-	11,651 ± 1373
	Procyanidin dimer B7	93%	C_30_H_26_O_12_	50	577.1357	42,121 ± 10,649	-
	Procyanidin trimer T3	73%	C_45_H_38_O_18_	50	865.1985	-	10,731 ± 1930
	Prodelphinidin dimer B3	80%	C_30_H_26_O_13_	50	593.1301	235,192 ± 24,370	193,398 ± 11,667
	Quercetin 3-*O*-glucuronide	91%	C_21_H_18_O_13_	50	477.0675	83,703 ± 6825	60,377 ± 3596
**Phenolic acids**	Gallic acid	90%	C_7_H_6_O_5_	50	169.0142	20,921 ± 1755	17,788.5 ± 1623
	Gallic acid 4-*O*-glucoside	92%	C_13_H_16_O_10_	50	331.0671	-	60,362 ± 11,976
	2,6-Dihydroxybenzoic acid	94%	C_7_H_6_O_4_	50	153.0193	-	5827 ± 957
	Ellagic acid	97%	C_14_H_6_O_8_	50	300.999	14,692 ± 4179	6479 ± 1558
	Sinapoyl glucose	90%	C_17_H_22_O_10_	50	385.114	242,036 ± 51,397	178,689 ± 1122
**Phenols**	Pyrogallol	86%	C_6_H_6_O_3_	50	125.0244	-	20,505 ± 120
**Other polyphenols**	3,4-Dihydroxyphenylglycol	80%	C_8_H_10_O_4_	50	169.0512	8176 ± 216	-
	Oleoside 11-methylester	93%	C_17_H_24_O_11_	50	403.1246	-	11,485 ± 3661
	Oleuropein	85%	C_25_H_32_O_13_	50	539.177	-	116,572 ± 33,774
	Resorcinol	91%	C_6_H_6_O_2_	50	109.0293	8290 ± 470	-
	Rosmadial	88%	C_20_H_24_O_5_	50	343.1549	22,938 ± 2316	-
	4-Vinylphenol	84%	C_8_H_8_O	50	119.0503	4643 ± 619	-
**Stilbenoids**	Resveratrol	85%	C_14_H_12_O_3_	50	227.0714	-	2746 ± 174
